# Different Families of Retrotransposons and DNA Transposons Are Actively Transcribed and May Have Transposed Recently in *Physcomitrium* (*Physcomitrella*) *patens*

**DOI:** 10.3389/fpls.2020.01274

**Published:** 2020-08-19

**Authors:** Pol Vendrell-Mir, Mauricio López-Obando, Fabien Nogué, Josep M. Casacuberta

**Affiliations:** ^1^ Centre for Research in Agricultural Genomics CSIC-IRTA-UAB-UB, Campus UAB, Edifici CRAG, Barcelona, Spain; ^2^ Department of Plant Biology, Swedish University of Agricultural Sciences, The Linnean Centre of Plant Biology in Uppsala, Uppsala, Sweden; ^3^ Institut Jean-Pierre Bourgin, INRAE, AgroParisTech, Université Paris-Saclay, Versailles, France

**Keywords:** *Physcomitrium (Physcomitrella) patens*, transposable element, transcription, genetic variability, centromere

## Abstract

Similarly to other plant genomes of similar size, more than half of the genome of *P. patens* is covered by Transposable Elements (TEs). However, the composition and distribution of *P. patens* TEs is quite peculiar, with Long Terminal Repeat (LTR)-retrotransposons, which form patches of TE-rich regions interleaved with gene-rich regions, accounting for the vast majority of the TE space. We have already shown that RLG1, the most abundant TE in *P. patens*, is expressed in non-stressed protonema tissue. Here we present a non-targeted analysis of the TE expression based on RNA-Seq data and confirmed by qRT-PCR analyses that shows that, at least four LTR-RTs (RLG1, RLG2, RLC4 and tRLC5) and one DNA transposon (*PpTc2*) are expressed in *P. patens*. These TEs are expressed during development or under stresses that *P. patens* frequently faces, such as dehydratation/rehydratation stresses, suggesting that TEs have ample possibilities to transpose during *P. patens* life cycle. Indeed, an analysis of the TE polymorphisms among four different *P. patens* accessions shows that different TE families have recently transposed in this species and have generated genetic variability that may have phenotypic consequences, as a fraction of the TE polymorphisms are within or close to genes. Among the transcribed and mobile TEs, tRLC5 is particularly interesting as it concentrates in a single position per chromosome that could coincide with the centromere, and its expression is specifically induced in young sporophyte, where meiosis takes place.

## Introduction

Mosses are one of the oldest groups of land plants, forming a sister clade with vascular plants (Leebens-Mack et al., 2019). Since the demonstration, in 1997, that gene targeting *via* homologous recombination was possible in *Physcomitrium* (*Physcomitrella*) *patens* (Schaefer and Zrÿd, 2001) this moss has become a leading plant model for answering essential questions in life sciences and in particular for understanding the evolution of biological processes of land plants. The draft of the *P. patens* genome was published in 2008 (Rensing et al., 2008), and a chromosome-scale assembly of the *P. patens* genome has been published (Lang et al., 2018), highlighting the similarities and differences with other plant genomes. Transposable Elements (TEs) account for the 57% of the 462,3 Mb of the assembled *P. patens* genome. This TE coverage is not very different from that of other plant genomes of similar size (Tenaillon et al., 2010). On the contrary, the distribution of TEs in *P. patens* is unusual as compared to other plants. TE-rich regions alternate with gene-rich regions all along the *P. patens* chromosomes (Lang et al., 2018) whereas in most plant genomes TEs accumulate in pericentromeric heterochromatic region on each chromosome. Interestingly, in spite of the general patchy TE distribution, a family of retrotransposons of the *copia* superfamily, RLC5 (comprised of full length, from now on RLC5, and truncated, tRLC5, elements), clusters at a single location in each chromosome that could correspond to the centromere (Lang et al., 2018). The TE-rich regions distributed all along the chromosomes are mainly composed of a single family of LTR-retrotransposons of the *gypsy* superfamily named RLG1 (Lang et al., 2018). RLG1 integrase contains a chromodomain, a type of protein domain that has been previously found To direct retrotransposon integration into heterochromatin (Gao et al., 2008), suggesting that RLG1 could target heterochromatic TE islands for integration. Although most TE copies are located in heterochromatic TE islands, gene-rich regions also contain some TE copies, with some of them that inserted recently and are polymorphic between the Gransden and Villersexel accessions (Lang et al., 2018). Moreover, the RLG1 retrotransposon is transcribed in *P. patens* protonema cells, suggesting that it can transpose during *P. patens* development (Vives et al., 2016; Lang et al., 2018). Although these data suggest that TE activity may have shaped the genome of *P. patens* and may continue to generate variability that potentially impact *P. patens* evolution, the global analysis of the capacity of *P. patens* TEs to be expressed and transpose is still lacking. Here we present an unbiased analysis of TE expression in *P. patens* based on RNA-Seq analyses and confirmed by qRT-PCR, that has allowed uncovering the developmentally or stress-related expression of different TE families, including class I (retrotransposons) and class II (DNA transposons) TEs. The data presented here reinforce the idea that TEs have shaped the genome of *P. patens* and show that they continue to drive its evolution.

## Materials and Methods

### RNA-Seq Data Used

RNA-Seq data were obtained from the *P. patens* Gene Atlas library (Perroud et al., 2018). In particular, we used RNA-Seq data obtained from stress-treated tissues (protoplasts, ammonium treatment, de- and rehydration, heat stress, and UV-B), different developmental stages, including protonemata in BCD, BCDA or in Knopp medium, protonemata in liquid and solid medium, gametophores, leaflets, and sporophytes (green and brown stages) and some hormonal treatments (Auxin, ABA or the Jasmonic acid precursor OPDA). A complete list of the data set used can be found in **Supplementary Table 1**.

### Transposable Element Transcriptome Assembly and Quantification

All selected reads where trimmed by quality using BBduk (https://sourceforge.net/projects/bbmap/). Reads mapping to the chloroplast, mitochondria or rRNA were discarded from the analysis. The remaining reads were mapped to the transposable element annotation (Hiss et al., 2017) using Bowtie2 (Langmead, 2013). All the reads that mapped were extracted using Samtools (Li et al., 2009). These reads were assembled to contigs using Trinity (Grabherr et al., 2011). In order to characterize and filter the assemblies, we aligned them to the TE library described in (Lang et al., 2018) using BLASTn (Altschul et al., 1990) with an e-value cutoff of 10^−5^. For transcripts corresponding to class I TEs, we kept only those showing alignments longer than 1000 nt. Manual inspection allowed discarding assemblies corresponding to poorly annotated TEs (i.e. repetitive genes like Leucine-Rich Repeat genes), solo LTR or chimeric TEs. The potentially coding domains of the selected assemblies were identified by a CDD-search, which allowed defining the orientation of the potentially expressed TEs (Marchler-Bauer et al., 2015).

In order to estimate the levels of expression of the elements corresponding to the selected assemblies, RNA-Seq reads were mapped to the selected assemblies using bowtie2 and only the reads potentially corresponding to sense transcripts were kept. To quantify the expression the number of mapping reads was normalized by the length of the assembly (Kb) and the total amount of trimmed reads for each condition without aligning the reads to the genome. The normalized expression data of each transcript and the sequence of the selected transcripts can be found in **Supplementary Table 1**.

### Plant Material

*P. patens* Gransden accession was used for all the samples used, with exception of the protonema vs sporophytes induction test where the *P. patens* Reute accession (Hiss et al., 2017) was used.

Protonemata were fragmented and plated on BCDAT medium overlaid with a cellophane disk in long-day conditions (16 h light 15 W m^−2^ to 8 h darkness) at 24°C for 7 days. Samples were collected at day 7 after 4 h of light. All the samples were frozen in liquid nitrogen immediately after harvesting and were kept at −80°C.

Protoplasts were isolated from 6 days old protonemal cultures after 30 min incubation in 1% driselase (Sigma D8037), 0.48 M mannitol. The suspension was filtered through two superposed sieves of 80 and 40 µm. Protoplasts where sedimented by low-speed centrifugation (600*g* for 5 min) and washed in 0.48 M mannitol.

The ABA treatment was performed as previously described (Perroud et al., 2018). Briefly, protonemal cultures were grown for 6 days on a cellophane disk on BCD medium. At day 6, the cellophane disks containing the protonemata tissues were transferred to BCD medium as control or to BCD containing 50 µM abscisic acid (Sigma A1049) for 24 h before harvesting.

Sporophyte RNA was obtained from Reute *P. patens*. Seven days old regenerated tissue from two consecutive rounds of a week old grinded material grown on solid BCDAT medium covered with cellophane was used as starting material. Six similar size small dots of moss tissue were plated in a 25 mm height petri dish (WVR international) containing BCD solid medium. They were grown for 40 days at 30 µmol m^−2^ s^−1^ constant white light regime and 25°C in a Sanyo MLR chamber. Then, plants were transferred to a Sanyo MLR chamber at an 8-h to 16-h light-dark cycle, 30 µmol m^−2^ s^−1^ light intensity and 15°C for reproductive gametangia induction and sporophyte development. After 20 days of post-reproductive induction (dpri), plants were submerged overnight in water to increase fertilization. Sporophyte samples were collected at 45 dpri showing a green round shape developmental stage. Each sporophyte was dissected under a Leica MZ16 stereomicroscope. Gametophyte tissue was discarded as much as possible and sporophyte was quickly frozen in liquid nitrogen. 40 dissected sporophytes were collected and used for RNA extraction.

### RNA Extraction and cDNA Production

Sporophyte RNA was obtained using the QIAGEN RNeasy mini kit following manufacturer’s protocol. DNA was removed by treating the samples with Ambion™ DNAseI kit (AM2222) following the manufacturer’s protocol. For all other tissues, RNA extraction and DNAse treatment was done using the Maxwell^®^ RSC Plant Kit (Promega). 500 ng of total RNA was used to synthetize the first-strand cDNA using the SuperScript™ III reverse transcriptase (Thermofisher).

### qRT-PCR

Quantitative real-time PCR were done in 96-well plates using the Roche LightCycler II instrument. SYBER Green I Master Mix (Roche Applied Science), primers at 1 µm and 1/20 dilution of the cDNA obtained from the reverse transcription were used for the qRT PCR. Each sample was run per triplicate with negative reverse transcriptase and non-template controls. The amplification conditions were: 95°C for 5 min, followed by 95°C for 10 s, 56°C for 10 s, and 72°C for 10 s, ending with the melting curve to check the specificity of the qRT-PCR. The housekeeping gene adenine phosphoribosyl transferase (APT) (Schaefer et al., 2010) was used to normalize the qRT-PCR results.

The primers used to check TE expression were designed using the Primer3plus software (Untergasser et al., 2012). The list of the primers used in this study can be found in **Supplementary Table S2**.

### Detection of Potentially Expressed TE Copies in the Genome and LTR-Retrotransposon Age Estimation

The TE copies most similar to the RNA assemblies, potentially representing the expressed elements, were identified by aligning the assemblies to the genome using Blastn with an e-value cutoff of 10E^−90^. However, in many cases the RNA assembly is obtained from the assembly of reads potentially generated by the expression of similar but different copies, and therefore, this approach may not be suitable. In order to identify the subset of elements potentially expressed in those cases, we also searched for elements showing a similarity of 80% over 80% of the sequence of the assembly. In those cases, we estimated the age of the subset of elements most similar to the assembled transcript and compared it to the age of all the complete elements of the same family annotated in the genome. To do that, we estimated first the Kimura two-parameter distance (Kimura, 1980) between the two Long Terminal Repeats (LTRs) and estimated the age using the formula T = K/2 × r, where T = time of divergence, K = divergence and r = substitution rate (Bowen and McDonald, 2001). Taking into account an estimated substitution rate of 9E−09 (Rensing et al., 2007).

### Transposable Element Polymorphisms Annotation

The publicly available DNA-seq resequencing data of three accessions of *P. patens* (Kaskaskia, SRX2234698; Reute, SRX1528135 and Villersexel, SRX030894) was used to look for TE polymorphisms with respect to the Gransden reference genome. Paired-end reads were mapped to the reference genome using BWA SW (Li and Durbin, 2009). TE insertions were detected using PoPoolationTE2 (Kofler et al., 2016) using the separate mode. To perform the analysis we kept only the non-reference insertions (insertions absent from the Gransden reference genome) predicted with a zygosity of at least 0.7. To establish the distance of these insertions to the closer genes the polymorphic TEs positions were intersected with that of the annotated genes using bedtools (Quinlan and Hall, 2010) using the function closestBed.

### Phylogenetic Analyses

To look for sequences similar to *P. patens* TEs in other genomes we first performed a blastn search against the complete NCBI nucleotide database. As this only retrieve sequences with significant similarity to RLG1 element we complemented this search with a blastx search of the *P. patens* TEs first against the complete NCBI non-redundant protein sequence database excluding *P. patens* and subsequently, in order to increase the chance to detect plant sequences, to the NCBI green plant database (taxid:33090). We performed the tblastx with the default parameters with a maximum target sequence of 250. The most similar sequence for each species was chosen as representative of the species. All the protein sequences were aligned using Mafft (Katoh and Standley, 2013) and trimmed using TrimAl (Capella-Gutiérrez et al., 2009). A phylogenetic tree was constructed using FastTree (Price et al., 2010) and visualized in iTOL (Letunic and Bork, 2019).

## Results

### A New Approach to Measure the Expression of *P. patens* Transposable Elements

**Figure 1:**
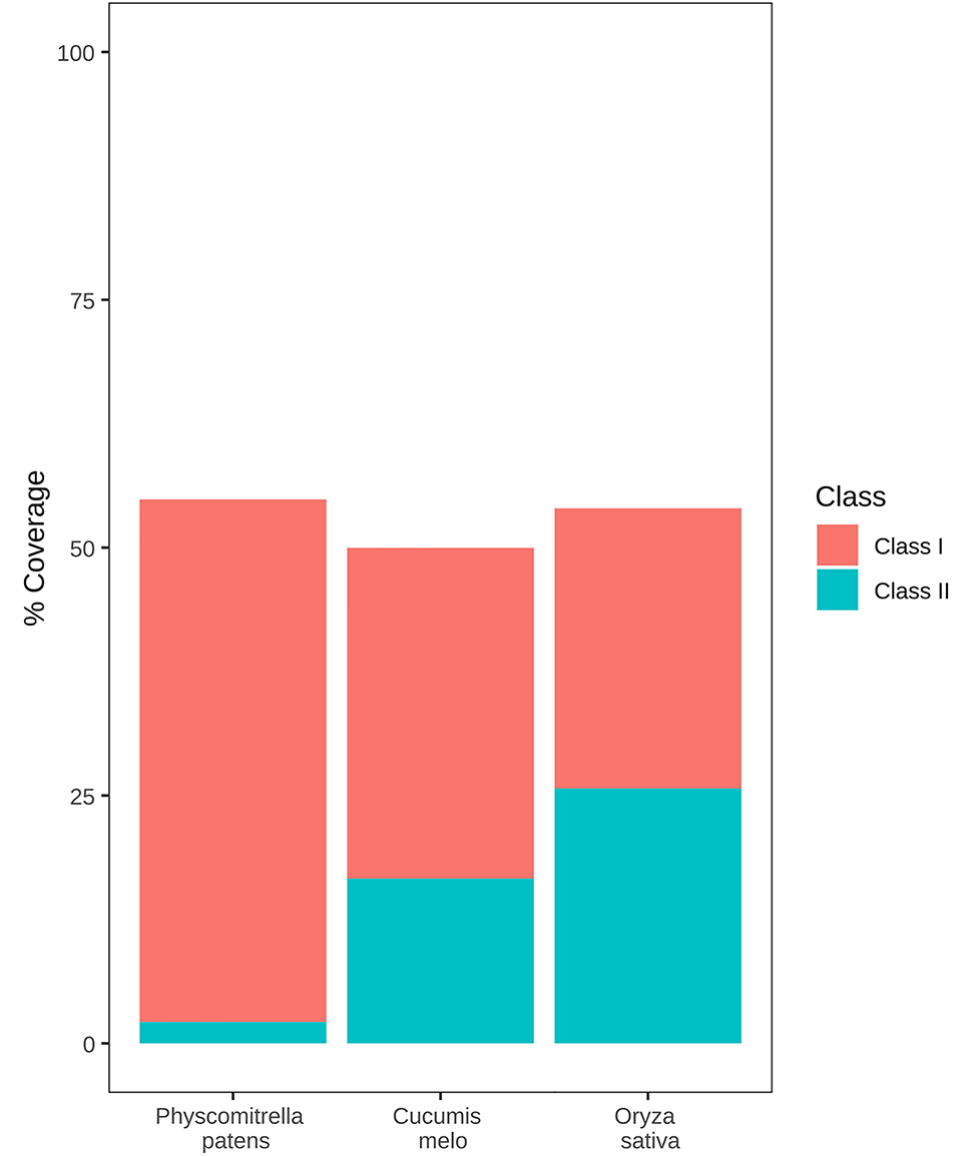
TE content of the *P. patens*, rice, and melon genomes. Genome coverage of class 1 and class 2 TEs is shown as red and blue boxes respectively.

More than half of the *P. patens* genome (57%) is occupied by TEs, a figure that is similar to that of other genomes of similar size (Tenaillon et al., 2010). As an example, the *P. patens* TE content is similar to that of two other genomes of similar sizes and for which the TE content has been annotated using the same REPET package (Flutre et al., 2011), such as rice (46.6%) (Ou et al., 2019) and melon (45,2%) (Castanera et al., 2020). However, *P. patens* has a very different TE composition as compared with these two genomes. Indeed, class II TEs account for 21.06% of the rice genome and 15.42% of the melon genome, in *P. patens* they only represent 6% of the genome (**Figure 1**). More strikingly, a single retrotransposon family, RLG1 accounts for almost half (47.44%) of the genome space occupied by class I elements (Lang et al., 2018). RLG1 is actively expressed in non-stressed protonema cells, and it may have transposed recently during *P. patens* evolution, as some of its copies are polymorphic between *P. patens* Gransden and Villersexel ecotypes (Vives et al., 2016; Lang et al., 2018).

RLG1 copies are concentrated in TE-rich heterochromatic islands and RLG1 transposition has therefore a limited capacity to induce gene variability. In order to explore the possibility that other TE families, apart from RLG1, could be expressed in particular developmental stages or stress situations, and could therefore generate new variability in gene regions, we took advantage of the large collection of *P. patens* RNA-Seq data available from the recently published *P. patens* gene atlas (Perroud et al., 2018), which includes data from different developmental stages and stress conditions. In addition of complete TEs, eukaryote genomes, and in particular those of plants, usually contain large amounts of defective and truncated elements that may be included in transcripts that are not the result of a genuine TE expression (Anderson et al., 2019). These transcripts can be sense or antisense with respect to the TE orientation and may in some cases participate in TE regulation, but cannot be considered as productive TE transcripts potentially involved in transposition. In *P. patens*, as it is common in eukaryote genomes and in particular in plants (Hoen et al., 2015; Bennetzen and Park, 2018), the fragmented and degenerated copies of TEs outnumber the complete and potentially functional copies. As a consequence, a quantification of the level of expression based on the number of RNA-Seq reads mapping to all TE-related sequences can lead to an overestimation of the expression of the different TE families. We have therefore decided to follow a strategy based on the detection of potentially complete transcripts obtained from an assembly of RNA-Seq reads, similar to what has previously been described for the analysis of the expression of human TEs (Guffanti et al., 2018). We used Trinity RNA-seq *de novo* assembly (Grabherr et al., 2011) to assemble reads showing similarity to annotated TEs (Lang et al., 2018). The 696 assemblies obtained were blasted back to the TE annotation to classify them. The vast majority (94%) of these 696 assemblies showed similarity to LTR-RT annotations, and an important fraction of them (72%) were short (less than 1000 nt) and corresponded to fragments of LTR-RTs, such as the LTRs. As an example, the assembly TRINITY_DN331_c0_g1 showed high sequence similarity to the LTR of RLC5 elements. A search for the genomic sequence most similar to that of the assembly identified a RLC5 solo-LTR located in the downstream proximal region of the Pp3c4_32070 gene annotation (**Supplementary Figure 1**). Interestingly, an analysis of the expression data available from the *P. patens* gene atlas (Perroud et al., 2018) showed that both the RLC5 solo-LTR and the Pp3c4_32070 annotated are specifically induced in gametophores treated with ABA, which strongly suggests that this solo-LTR is expressed as a consequence of read-through transcription from the gene promoter. In order to eliminate assemblies corresponding to the expression of fragments of LTR-RTs, and taking into account that typical complete LTR-RTs are several kb long, we discarded all the LTR-RT assemblies shorter than 1,000 nt. The remaining 172 transcripts were analyzed for the potential presence of regions coding for the typical class I and class II TE protein domains and their alignments to annotated TE sequences were manually inspected to discard those showing similarities to poorly annotated transposable elements, and truncated or chimeric elements. As an example, **Supplementary Figure 2** shows the analysis of TRINITY_DN99_c0_g1_i5 that corresponds to a complex region containing different degenerated TE fragments that seem to be transcribed as a single transcription unit. Among the 22 assemblies retained, some corresponded to the antisense strand of annotated TEs. After manual inspection, some of these were shown to correspond to LINE elements (see **Supplementary Figure 3** for an example). These transcripts may participate in the control (e.g. silencing) of TE expression but cannot be considered as genuine TE transcription. The assemblies corresponding to potential antisense transcripts were discarded. An analysis of the remaining assemblies showed that they corresponded to 9 different potentially complete annotated TEs and were selected for further analysis.

### Both Retrotransposons and DNA Transposons Are Expressed in *P. patens*

The analysis of the transcript assemblies showed that they correspond to 9 different *P. patens* TEs: 2 LTR retrotransposons of the *gypsy* superfamily (RLG1 and RLG2), two of the *copia* superfamily (RLC4 and RLC5), with one of them potentially corresponding to the two different forms of RLC5, the full-length and the truncated form (RLC5/tRLC5) and two different DNA TEs belonging to the Mariner superfamily, that were not properly annotated in the *P. patens* TE annotation (Lang et al., 2018), but had been previously identified as *PpTc1* and *PpTc2* (Liu and Yang, 2014). In addition, the manual inspection of the alignments of the transcript assemblies with the annotated TEs allowed refining the annotation of two elements annotated as unclassified non-LTR retrotransposon that we could identify as a potentially expressed complete LINEs (LINE-1 and LINE-2). The RNA-Seq reads obtained from the RNAs generated by the expression of a TE family show a certain degree of sequence variability, and therefore, they are not all of them identical to the assembly that represents the complete RNA of the family. On the other hand, this assembly is in most cases not identical to any of the of the TE copies of that particular TE family. This suggests that, for most TE families, different elements are concomitantly expressed and that the RNA assembly should be considered as a consensus of the expressed RNAs.

These results suggest that different families of both retrotransposons and DNA transposons are transcribed in *P. patens.*

#### *P. patens* Contains TEs Closely Related to Fungal TEs

**Figure 2:**
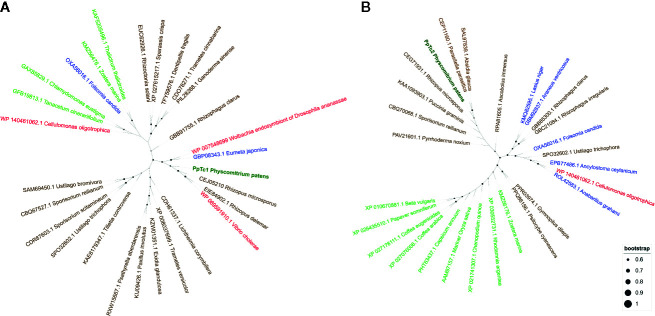
Phylogenetic analysis of the transposases potentially encoded by *PpTc1* **(A)** and *PpTc2* **(B)** with those potentially encoded by plant, fungal, animals and bacterial Mariner-like elements. *P. patens* sequences are shown in dark green, plant sequences in light green, fungal sequences in brown, animal sequences in blue and bacterial sequences in red.

A preliminary characterization of the two Mariner-like elements found to be potentially expressed suggested that these elements were different from other plant Mariner-like elements, they being more closely related to fungal TEs of the Mariner superfamily. As this result was somehow surprising, we searched for sequences potentially corresponding to transposases of similar elements in the phylogenetically related liverwort *Marchantia polymorpha* and in well-characterized dicotyledonous and monocotyledonous plants such as Arabidopsis and rice. These searches did not retrieve significant hits, suggesting that these genomes do not contain sequences related to Mariner-like elements similar to those found in *P. patens*. A phylogenetic analysis of the potential transposases of Mariner-like sequences present in public databases more similar to those of the two Mariner-like elements found in *P. patens*, and including other Mariner-like sequences from plants, shows that the *P. patens* elements are closely related to elements found in fungal genomes, and are not related to *Marchantia polymorpha* or other plant sequences (**Figure 2**). These results may indicate a horizontal transfer of these TEs from fungi. In order to explore whether other TEs may have also experienced a similar phenomenon, we extended the phylogenetic analysis performed for the two Mariner-like elements to the other *P. patens* TE families here described. These analyses showed that, in contrast to what happens for the two Mariner-like elements, databases contain plant sequences with significant similarity to the rest of TE families here described. However, the phylogenetic analyses performed show that whereas the trees obtained for *P. patens* RLG2, RLC4, LINE-1 and LINE-2 retrotransposons are congruent with the phylogenetic relationships of the species, this is less obvious for RLG1 and tRLC5 (**Supplementary Figures 4–8**). This may suggest that, in addition to the two Mariner-like elements, other *P. patens* TEs may have been transferred horizontally from fungal species.

### Developmental and Stress-Related Expression of *P. patens* TEs

**Figure 3:**
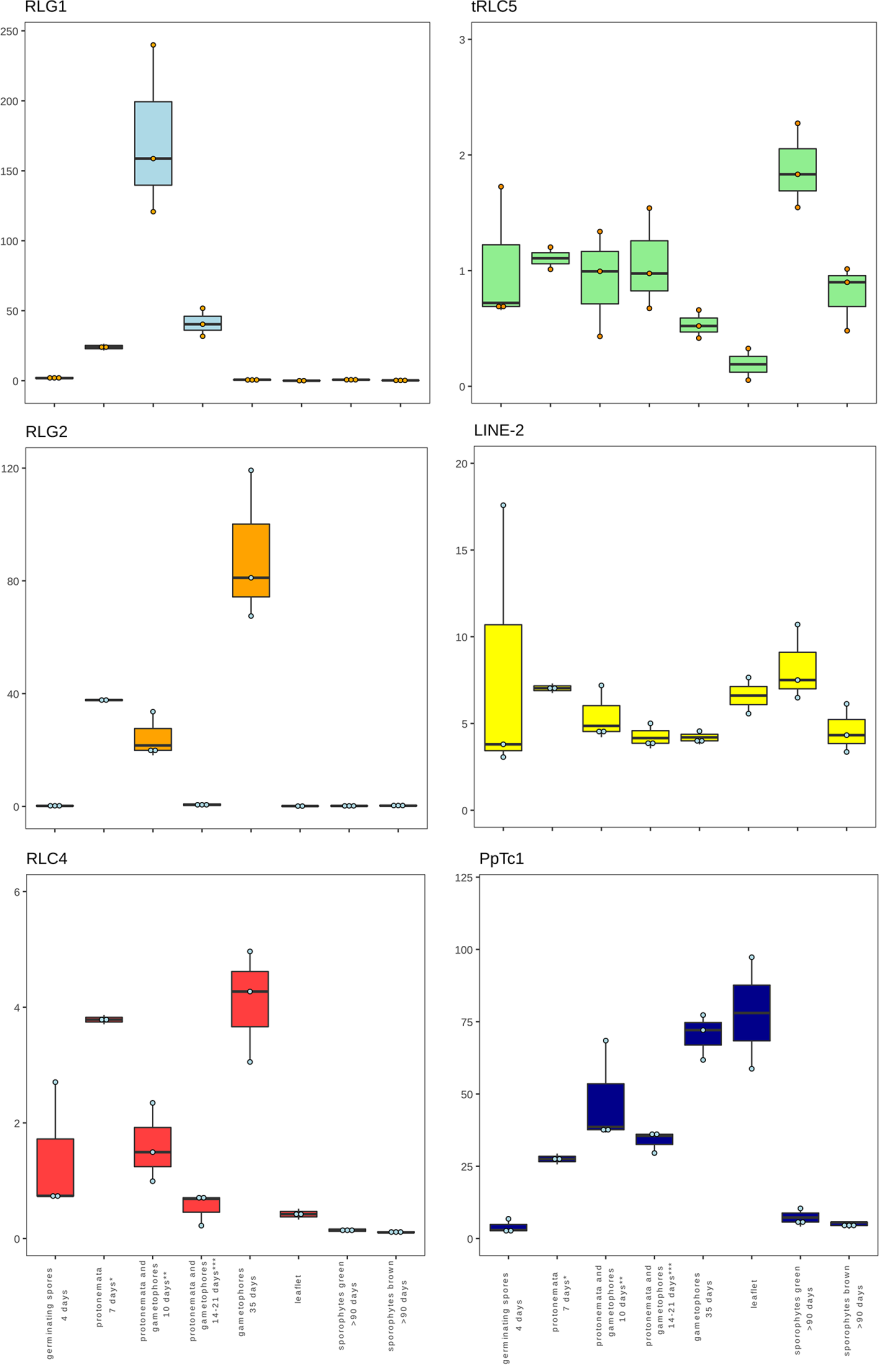
Developmental expression of *P. patens* TEs. Normalized TE expression (see methods) in different developmental conditions selected from the *P. patens* Gene Atlas library (Perroud et al., 2018).

**Figure 4:**
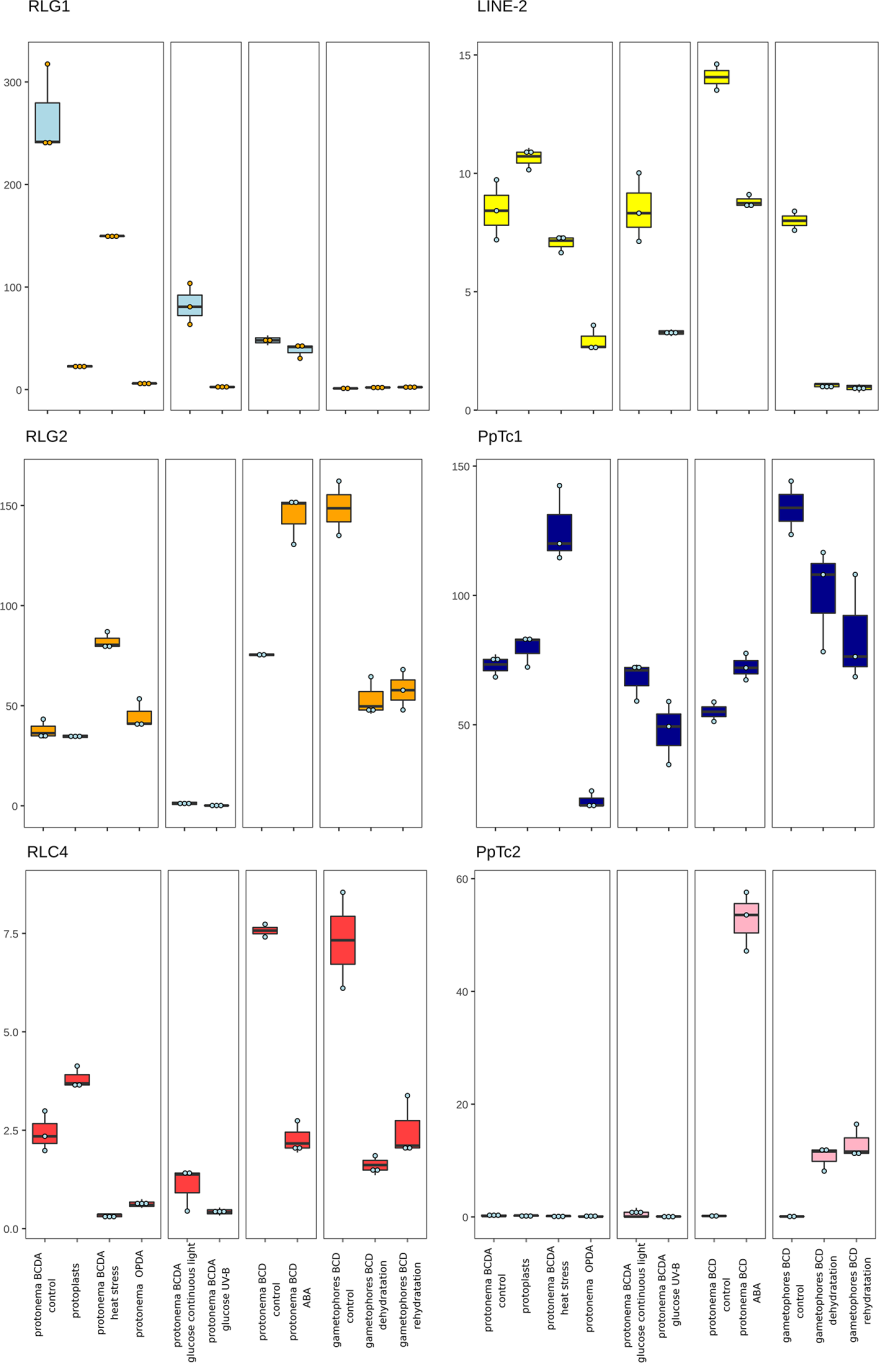
*P. patens* TE expression under stress conditions. Normalized TE expression (see methods) under different stress conditions selected from the *P. patens* Gene Atlas library (Perroud et al., 2018).

**Figure 5:**
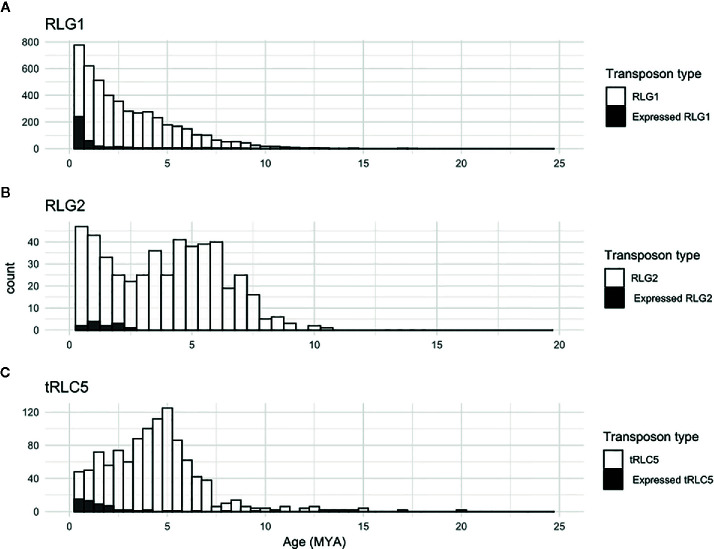
Relative age of expressed TEs. Kimura-2-parameter distance between the two LTRs of all elements (white bars) or of elements similar to the corresponding RNA assembly, and therefore potentially expressed (black bars) belonging to the RLG1 **(A)**, RLG2 **(B)** and tRLC5 **(C)** families.

The availability of RNA-Seq data from different developmental stages and stress conditions (Perroud et al., 2018) allowed us to perform and unbiased analysis of the patterns of expression of the different transcribed *P. patens* TEs. We have previously shown that RLG1 is expressed in non-stressed protonema cells and its expression is reduced in protonema-derived protoplasts. RLG1 seems thus to be repressed by stress, in clear contrast with the stress-related expression of most TEs, as already discussed (Vives et al., 2016; Lang et al., 2018). Here we confirm that RLG1 is expressed in protonema, its expression increasing as the protonema develops and decreasing when gametophores develop, and is repressed in protoplasts (**Figures 3** and **4**). On the other hand, RLG1 does not seem to be expressed in other tissues and it is repressed by several of the stresses analyzed, in particular by heat shock and UV-B light (**Figures 3** and **4**). We confirmed the RLG1 expression in protonema cells and its repression in protonema-derived protoplasts by qRT-PCR (**Supplementary Figure 9**). A comparison of the RLG1 assembled RNA with all the RLG1 genomic copies suggests that only a subset of the RLG1 elements is expressed (**Table 1**). An analysis of the putative ages of these elements, by analyzing the sequence differences between the two LTRs of each element, suggests that only the youngest RLG1 elements are transcribed (**Figure 5A**).

**Table 1:** Total number of complete elements (total), number of elements showing 80% identity over 80% of the length of the corresponding RNA assembly (80/80) for each indicated TE family.

	Total	80/80
RLG1	5092	3636
RLG2	529	25
RLC4	96	75
tRLC5	332	88
PpTc2	22	22

RLG1 is the TE expressed at the highest level in *P. patens* but, as already mentioned, we show here that other TEs are also expressed during *P. patens* development or under particular environmental conditions. The second Gypsy-like LTR-RT family found to be expressed, RLG2, is also expressed in protonema cells, and its expression increases in gametophores (**Figure 3**). On the other hand, the expression of RLG2 is strongly induced by ABA and heat stress in protonema, and repressed when gametophores are submitted to dehydratation and rehydratation (**Figure 4**). We confirmed the induction of RLG2 expression by ABA by qRT-PCR analyses (**Supplementary Figure 10**). Similarly, to RLG1, the comparison of the RLG2 assembled RNA with the RLG2 genomic copies shows that only the youngest RLG2 elements are transcribed in the conditions tested (**Table 1** and **Figure 5B**).

The two *copia* retrotransposon families found here to be expressed, show low levels of expression during *P. patens* development. RLC4 seems to be particularly expressed in gametophores, whereas tRLC5 seems to be more expressed in sporophytes. RLC4 expression seems to be repressed in most stress conditions, although the levels of expression are very low in all cases.

tRLC5 is a particularly interesting family of TEs, as tRLC5 copies have been proposed to mark the centromere and participate in the centromeric function (Lang et al., 2018). The data presented suggest that tRLC5 may be particularly expressed in green sporophytes (**Figure 3**). In order to confirm this pattern of expression we performed qRT-PCR experiments. As the Gransden ecotype produces few sporophytes, which makes it difficult to analyze sporophyte-specific expression, we used Reute tissues, as this ecotype produces many more sporophytes in laboratory conditions (Hiss et al., 2017). This analysis confirmed that tRLC5 expression is induced in young sporophytes (**Supplementary Figure 11**). A comparison of the tRLC5 assembled RNA with the tRLC5 genomic copies suggests that only the youngest tRLC5 elements are transcribed (**Table 1** and **Figure 5C**).

LINE-1 seems to be expressed at a very low level in all conditions and we have not detected any relevant change in expression upon stress (not shown). On the other hand, LINE-2 is also expressed at a low level in most tissues but shows an increased expression in sporophytes and germinating spores (**Figure 3**). A comparison of the LINE-2 assembled RNA with the genomic copies suggests that the expressed LINE-2 is located in the close vicinity of an annotated gene (Pp3c16_3270) and the mapping of the RNA seq reads to this region suggests that LINE-2 could be expressed as the result of a readthrough transcription of this gene (**Supplementary Figure 12**). Indeed, although there are some minor differences, the patterns of expression of Pp3c16_3270 and LINE-2 during development or under particular stress conditions are mostly coincident (not shown).

Finally, of the two Mariner-like elements analyzed, only *PpTc1* is expressed in non-stressed tissues, with a particularly high expression in gametophores and leaflets (**Figure 4**), but both *PpTc1* and *PpTc2* are strongly induced by stress. *PpTc1* expression is particularly induced by heat stress, whereas *PpTc2* is only expressed after ABA induction or after dehydration or rehydration of gametophores (**Figure 4**). A comparison of the two Mariner-like assembled RNAs with their genomic copies identified the two elements potentially transcribed. Both elements are located close to a gene, and the analysis of the patterns of expression of both genes provides information on the possible expression of the two Mariner-like elements. In the case of *PpTc1* the TE is only expressed in the conditions where the gene (Pp3c20_23510V3.1) is expressed (**Supplementary Figure 13**), which suggests that the expression detected for *PpTc1* could be the result of read-through transcription from the neighboring gene. On the contrary, the expression of *PpTc2* and the gene located nearby (Pp3c9_17220V3.1) do not overlap. Indeed, only *PpTc2*, and not the gene located nearby, is expressed in gametophores submitted to dehydration and rehydration and its expression is strongly induced in protonema treated with ABA which is not the case for the close by gene (**Supplementary Figure 14**). We confirmed the induction of *PpTc2* by ABA by qRT-PCR (**Supplementary Figure 15**). Therefore, whereas we cannot rule out the possibility that *PpTc1* expression could be the result of a readthrough expression from a neighboring gene, the transcript corresponding to *PpTc2* seems to be the result of a genuine TE transcription. Moreover, the sequence variability of the RNA-Seq reads corresponding to *PpTc2*, suggests that other *PpTc2* elements may also be expressed. Indeed, although the *PpTc2* copy located in the vicinity of the Pp3c9_17220V3.1 gene is almost identical to the RNA assembly (99.4%), other PpTc2 copies also show high similarity to the assembly (**Table 1**) and may also be expressed.

### TE Mobility During Recent *P. patens* Evolution

The transcription of a copy of the TE in case of retrotransposons, and/or of the proteins necessary to mobilize the element, is the first and obligatory step of TE transposition. Therefore, the transcription of the different TEs reported here suggests that different TEs may have recently moved during *P. patens* evolution. We have already reported that this is indeed the case for RLG1, as RLG1 elements are polymorphic between the Gransden and Villersexel accessions. Here we decided to expand the analysis for possible insertion polymorphisms to all *P. patens* TEs using data from 4 different *P. patens* accessions, Reute, Kaskaskia, Villersexel, and the one from which the reference genome has been obtained, Gransden. To this end we used PopoolationTE2 to look for TE polymorphisms among these accessions using paired-end short-read resequencing data from Reute, Kaskaskia, Villersexel, that we mapped to the Gransden reference genome.

We found an important number of RLG1 polymorphisms in the three analyzed accessions with respect to Gransden (**Table 2**). The number of polymorphisms in Reute was much smaller than in the two other accessions, which is in accordance with the close genetic relationship between Gransden and Reute (Hiss et al., 2017). Interestingly, in addition to polymorphisms related to RLG1 elements, we also detected polymorphic insertions of RLG2, RLG3, tRLC5/RLC5 and *PpTc1* (**Table 2**). In general, the number of polymorphisms is higher in Villersexel and smaller in Reute, as seen for RLG1.

**Table 2:** TE polymorphisms in the different *P. patens* accessions.

Accession	RLG1	RLG2	RLG3	RLC4	tRLC5/RLC5	LINE-1	LINE-2	PpTc1	PpTc2	Total
Reute	18	0	4	0	5	0	0	0	0	27
Kaskaskia	147	0	15	0	17	0	0	0	0	179
Villersexel	229	2	48	2	21	0	0	1	0	303
Total	394	2	67	2	43	0	0	1	0	509

The number of polymorphic insertions was particularly high for RLG3 and tRLC5/RLC5. In order to start analyzing the potential impact of the polymorphic insertions described here in the phenotypic differences between the four *P. patens* ecotypes, we analyzed the locations of the polymorphic TE insertions (**Supplementary Table 3**) and found that 20% of them are located close to genes, with potential consequences on their coding capacity or expression (**Table 3**).

**Table 3:** Distance of polymorphic TE insertions to genes.

Accession	Inside Genes	< 1 kb closest gene	> 1 kb closest gene	Total
Reute	4	4	19	27
Kaskaskia	12	27	140	179
Villersexel	22	34	247	303
Total	38	65	406	509

## Discussion

### The Challenging Analysis of TE Transcription

Different programs to measure TE transcription from NGS data exist (Jin et al., 2015; Lerat et al., 2017). These programs usually rely on mapping RNA-Seq reads to a TE annotation or a consensus of a TE family. Although these programs can be very useful for certain genomes and particular TE families, they may not be adequate in others. Indeed, most eukaryote genomes, and in particular those of plants, contain an important number of fragmented or degenerated TE copies in addition to full copies of TEs. As the TE fragments can also be included in transcripts, and outnumber the complete copies (Hoen et al., 2015; Bennetzen and Park, 2018), an estimation of the expression of TEs that would not discriminate between transcripts corresponding to TE fragments or to complete elements will overestimate the expression of certain families and will lead to erroneous results. This is what we came across when starting to analyze the expression of *P. patens* TEs. As an example, as already explained, among the short assemblies discarded there was one (TRINITY_DN331_c0_g1) corresponding to a RLC5 solo-LTR. An analysis of the RNA-Seq reads matching this assembly showed their specific accumulation in ABA-treated protonema cells and in gametophores under dehydration/rehydration stress. The results presented here show that the RLC5 solo-LTR is expressed as the result of read-through transcription from the ABA-induced Pp3c4_32070 gene located just upstream of it. An analysis of RLC5 expression based solely on mapping RNA-Seq reads to the TE annotation would have led to the wrong conclusion that RLC5 is induced by ABA and drought stresses. On the contrary, the approach described here, which is similar to the one previously described for the analysis of the expression of human TEs (Guffanti et al., 2018), allows for the assessment of the expression of RNAs corresponding to complete elements potentially resulting from genuine TE transcription.

### Different Retrotransposon and DNA Transposon Families Are Transcribed in *P. patens*

The results presented here show that at least four LTR-RTs (RLG1, RLG2, RLC4 and tRLC5) and one DNA transposon (*PpTc2*) are expressed in *P. patens*. Among those, RLG1 and RLG2 are highly expressed during normal *P. patens* development, RLG1 being expressed mainly in protonema tissues whereas the expression of RLG2 is increased in gametophores. RLC4 seems also to be expressed in gametophores, albeit at a low level, and tRLC5 is expressed in young sporophytes. Therefore, during *P. patens* development, there is an important expression of different transposons. In addition, although RLG1 seems to be repressed by most stresses, different TEs are activated by stress. RLG2 is overexpressed under heat shock and ABA treatment, and *PpTc2* is induced by ABA and by dehydration and rehydration treatments. Mosses are known to be tolerant to dehydration and rehydration (Cuming et al., 2007; Cui et al., 2012), which, together with the associated changes of temperature, are part of their natural lifestyle. The dehydration/rehydration stresses and the ABA treatment, known to mediate the responses to those stresses (Cuming et al., 2007), and to some extent heat stress, could thus be considered as part of the normal development of *P. patens* or, at least, frequent stresses *P. patens* has to face.

### Recent Mobilization of *P. patens* TEs

The expression of different TEs in normal *P. patens* growing conditions could allow the mobilization of TEs and the generation of genetic variability that could potentially affect gene expression/function in this haploid species. The analysis presented here shows that many TE insertions are polymorphic between different *P. patens* accessions. Indeed, we have detected an important number of polymorphic insertions of RLG1, RLG3 and tRLC5/RLC5 elements. The high number of polymorphisms related to RLG3 is intriguing as we did not detected expression. RLG3 may therefore be expressed under different environmental conditions not tested here. Alternatively, RLG3 may have lost the ability to transcribe and transpose recently during evolution. In all cases, the highest number of polymorphisms with respect to the Gransden accession is found in Villersexel and the lowest in Reute, which is in accordance with the number of SNPs these accessions show with respect to the Gransden reference genome (Lang et al., 2018). We have also found a small number of polymorphic insertions of RLG2, RLC4 and *PpTc1*. The number of detected TE polymorphisms with respect to the Gransden reference genome in these accessions is probably underestimated, as none of the programs available to look for TE polymorphisms, including the one used here, can detect polymorphic TE insertion sitting in repetitive regions (Vendrell-Mir et al., 2019). In any case, the polymorphisms detected here illustrate the potential of TEs to generate genetic variability in *P. patens*. Moreover, an important fraction of the polymorphisms detected are within or close (less than 1 Kb) to a gene, which suggests that TE movement may have impacted gene coding or gene regulation, and therefore may have contributed to the phenotypic variability of *P. patens*.

### The Heterochromatic tRLC5 Elements Are Transcribed in Sporophytes

In addition to generate new alleles or new gene regulations, TEs are also involved in chromosome structure and function. In plants, TEs have been shown to provide origins of replication in heterochromatic regions (Sequeira-Mendes et al., 2019), and are frequently part of centromeres (Lermontova et al., 2015). Different retrotransposon have been found to specifically accumulate in the centromeres of the green algae *Coccomyxa subellipsoidea* (Blanc et al., 2012) or the liverwort *M. polymorpha* (Diop et al., 2020) were they could support centromere function. Interestingly, tRLC5 was previously proposed to mark the centromere and participate to the centromere function in *P. patens* (Lang et al., 2018). We show here that tRLC5 is transcribed in *P. patens*. In spite of its heterochromatic nature, centromere sequences have been shown to be transcribed in yeast, animals and plants and this transcription seems vital for the maintenance of the centromere chromatin identity and in several aspects of centromere function (Chan and Wong, 2012; Perea-Resa and Blower, 2018). Young sporophytes are a key developmental stage of *P. patens* where meiosis takes place (Charlot et al., 2014). We show here that most meiosis-specific genes (Mercier et al., 2015) are highly induced in green sporophytes (**Supplementary Figure 16**), the developmental stage where tRLC5 is expressed. It has been proposed that demethylation of centromeric DNA during meiosis may allow the transcription of centromeric sequences, which could serve as markers recognized by other factors and allow centromere assembly (Liu et al., 2015). The expression of tRLC5 in the centromere, at the moment meiosis takes place, could thus play a role in centromere assembly and function during this key process. On the other hand, the transcription pattern of tRLC5, specifically activated in young sporophytes, is reminiscent of the expression of the Athila retrotransposon of Arabidopsis, which also concentrates in the centromere and is expressed in the pollen grain (Keith Slotkin, 2010). It has been proposed that TE expression in the vegetative nurse cells of the pollen may allow re-establishing its silencing in the sperm cells (Martínez et al., 2016). The expression of tRLC5 in the sporophyte could also fulfill a similar role. Further experimental work will be required to explore any of these two non-exclusive hypotheses.

### Are Some of the *P. patens* TE Families the Result of a Horizontal Transfer from Fungal Species?

In addition to the characterization of the transcriptional activity of *P. patens* TEs, the work presented also allowed us to better characterize two Mariner-like elements. These *P. patens* elements, that are transcribed and mobile, are more closely related to fungal elements than to any Mariner-like element found in plants, suggesting that they may have been horizontally transmitted from fungi. Interestingly, another *P. patens* Mariner-like element already described was also shown to be closely similar to fungal TEs (Castanera et al., 2016), which suggest that the horizontal transfer of Mariner-like elements from fungi to *P. patens* may have been a frequent event. The Mariner TE family is ubiquitous in the genomes of virtually all extant eukaryotic species and seem to be particularly prone to horizontal transfer, probably because they contain a transcriptionally promiscuous “blurry” promoter (Palazzo et al., 2019). Early land plants were aided by mutualistic interactions with fungi and these symbiotic interactions with fungi have been maintained in some bryophytes such as *M. polymorpha* (Humphreys et al., 2010). Surprisingly, although *P. patens* contains the strigolactone signaling pathway, which induce mycorrhizal signaling, it has not been shown to establish mycorrhizal interactions (Delaux et al., 2013; Field and Pressel, 2018; Rensing, 2018). The potential horizontal transfer of Mariner-like elements could be a remnant of this lost interaction, although an ulterior close contact between *P. patens* and different fungi may have also be at the origin of these horizontal transfers. It is interesting to note that *P. patens* is the only plant that shares with fungi the traces of past infections of giant virus relatives (Maumus et al., 2014), which also highlights the close relationship with fungi that *P. patens* has maintained during its recent evolution.

## Conclusion

In summary, the results presented here show that TEs have an important activity in *P. patens*, with the transcriptional activation of different TE families in normal *P. patens* growing conditions, suggesting that TEs may have shaped *P. patens* genome and may continue to contribute to its function, including adaptation to stresses and the intraspecific genetic variability.

## Data Availability Statement

All datasets generated for this study are included in the article/**Supplementary Material**.

## Author Contributions

JC and FN conceived the project. PV-M performed all the experiments. ML-O obtained the RNA from green sporophytes. PV-M, FN, and JC drafted the manuscript. All authors contributed to the article and approved the submitted versión.

## Funding

This work was supported by grants from the Ministerio de Economia y Competitividad (AGL2016-78992-R) to JC and from the Investissement d’Avenir program of the French National Agency of Research for the project GENIUS (ANR-11-BTBR-0001_GENIUS) to FN. The work at CRAG is supported by a Spanish Ministry of Economy and Competitivity grant for the Center of Excellence Severo Ochoa 2016–2019 (SEV-2015-0533); the IJPB benefits from the support of Saclay Plant Sciences-SPS (ANR-17-EUR-0007). PV-M holds a FPI (Formación de Personal Investigador) fellowship from the Spanish Ministerio de Economia y Competitividad.

## Conflict of Interest

The authors declare that the research was conducted in the absence of any commercial or financial relationships that could be construed as a potential conflict of interest.
