# Supplementary material for: Different Families of Retrotransposons and DNA Transposons Are Actively Transcribed and May Have Transposed Recently in Physcomitrium (Physcomitrella) patens
Source: Front Plant Sci. 2020 Aug 19;11:1274. doi: 10.3389/fpls.2020.01274 (PMC7466625; doi:10.3389/fpls.2020.01274)
Supplement: Supplementary file 1 [file DataSheet_1.docx]

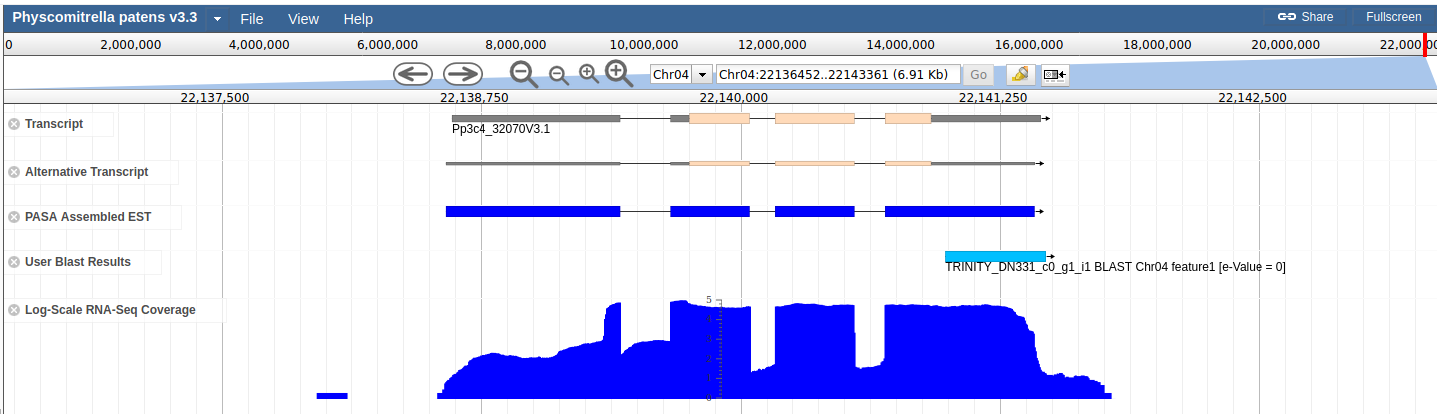


**Supplementary Figure 1: Expression of a RLC5 solo-LTR as the result of a read-through transcription from the gene Pp3c4_32070.** Screenshot of the Phytozome genome browser showing from top to bottom, the Pp3c4_32070 transcript, alternative transcripts, PASA Assembled EST, BLASTn best-hit of the TRINITY_DN331_c0_g1_i1, corresponding to a RLC5 solo-LTR, and the log-scale RNA-Seq coverage of the genome region.


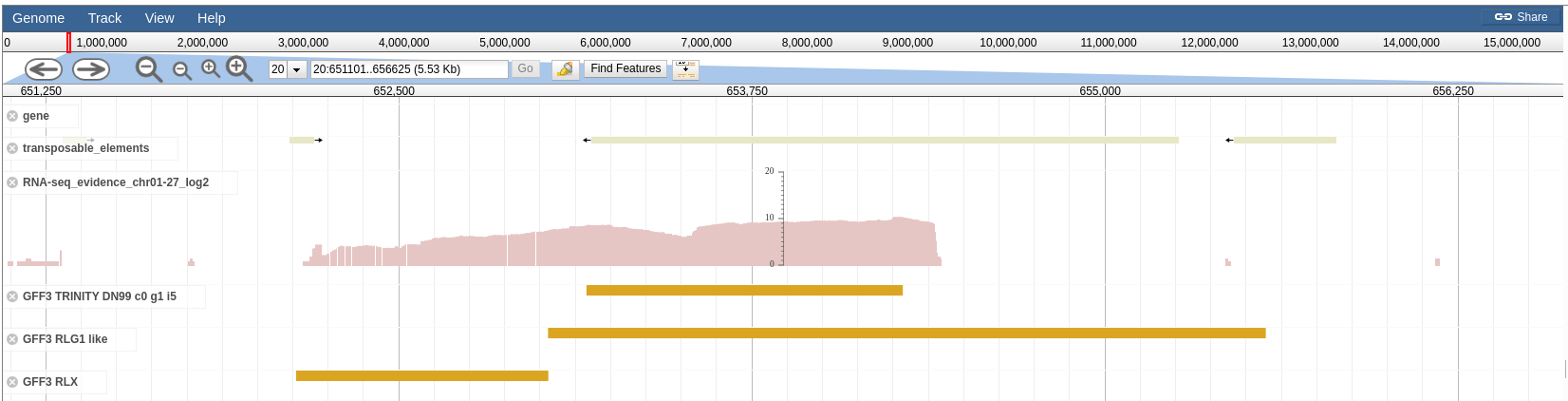


**Supplementary Figure 2: Expression of a chimeric TE sequence.** Screenshot of the COGE https://genomevolution.org/coge/) genome browser showing from top to bottom, the annotated genes in the genomic region, the TE annotation corresponding to a RLGU (unclassified Gypsy) element, the log-scale RNA-Seq coverage of the genome region, the best hit of the assembly TRINITY_DN99_c0_g1_i5, the manually curated truncated RLG1 element, and a manually curated repetitive sequence corresponding to a copy of an RLX (LTR-retrotransposon unclassified).


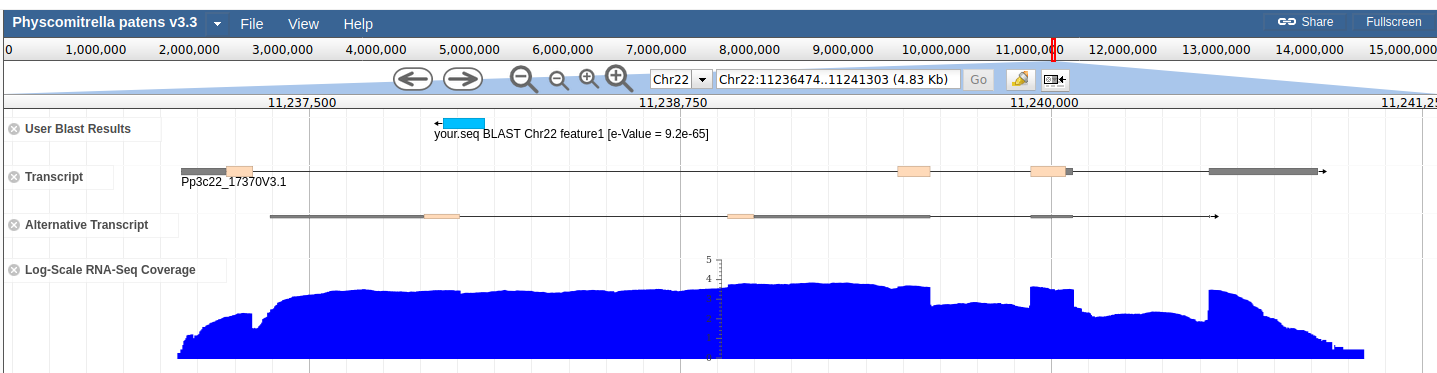


**Supplementary Figure 3: Antisense expression of a LINE inserted within an intron as a result of gene expression.** Screenshot of the Phytozome genome browser showing from top to bottom, the best hit of the assembly TRINITY_DN734_c0_g1_i1, the Pp3c22_17370 gene annotation, the alternative transcripts, and the log-scale RNA-Seq coverage of the genome region.


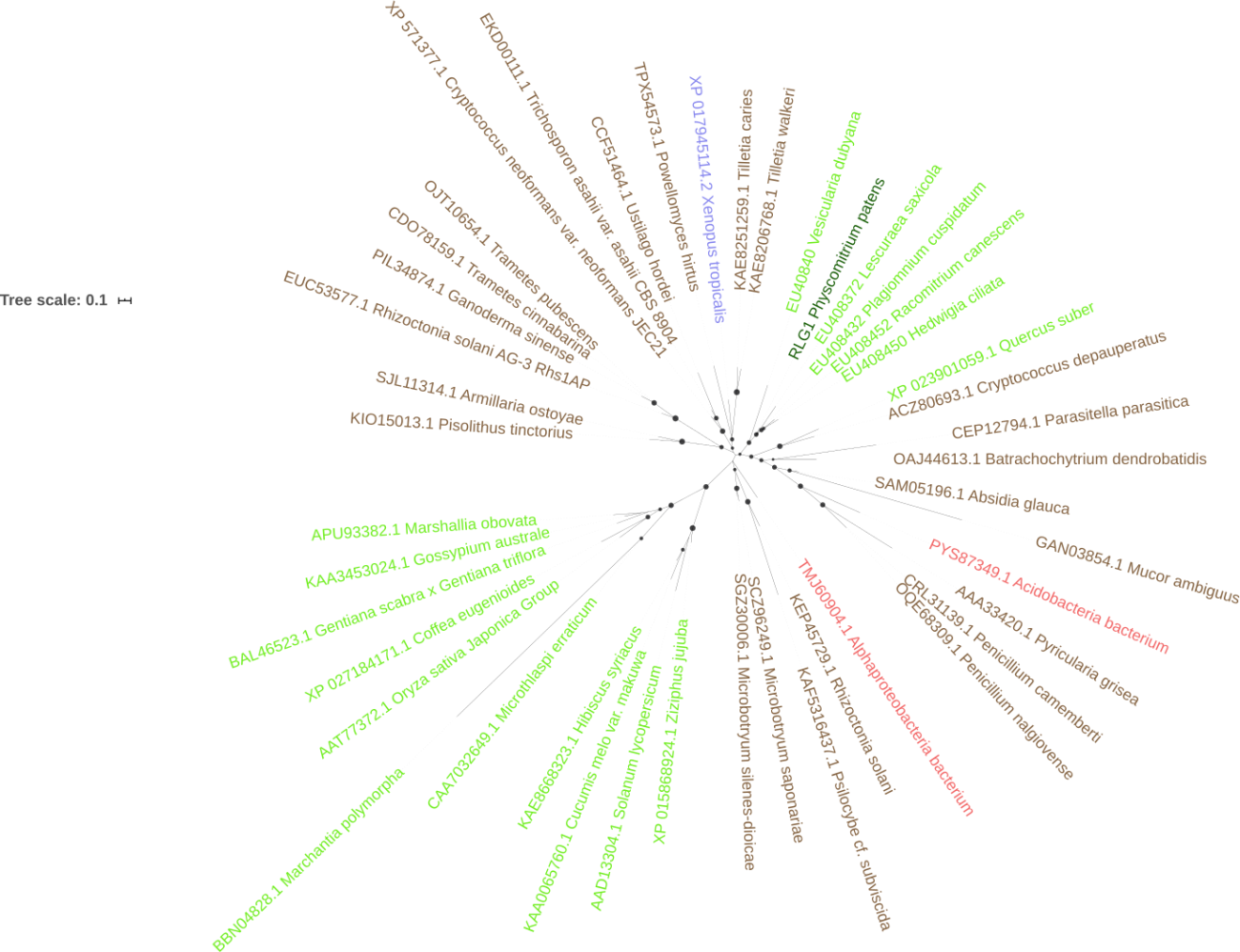

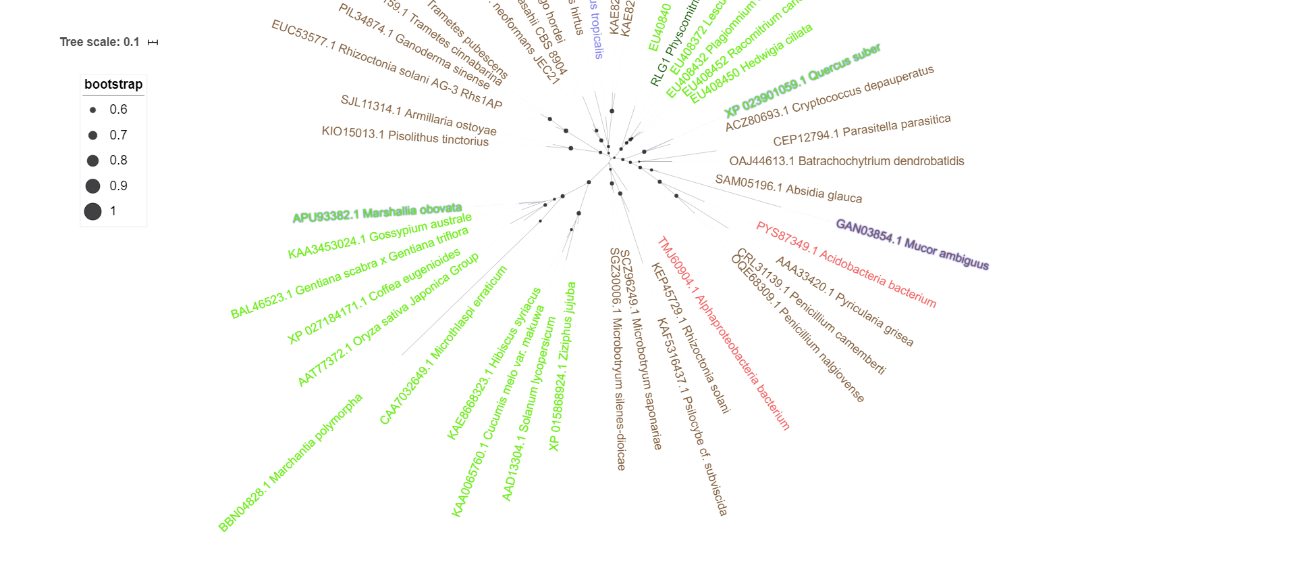


**Supplementary Figure 4: Phylogenetic analysis of the Transposable elements RLG1 proteins with those potentially encoded by plant, fungal, animals and bacterial transposable elements elements.** *P. patens* sequences are shown in dark green, plant sequences in light green, fungal sequences in brown, animal sequences in blue and bacterial sequences in red.


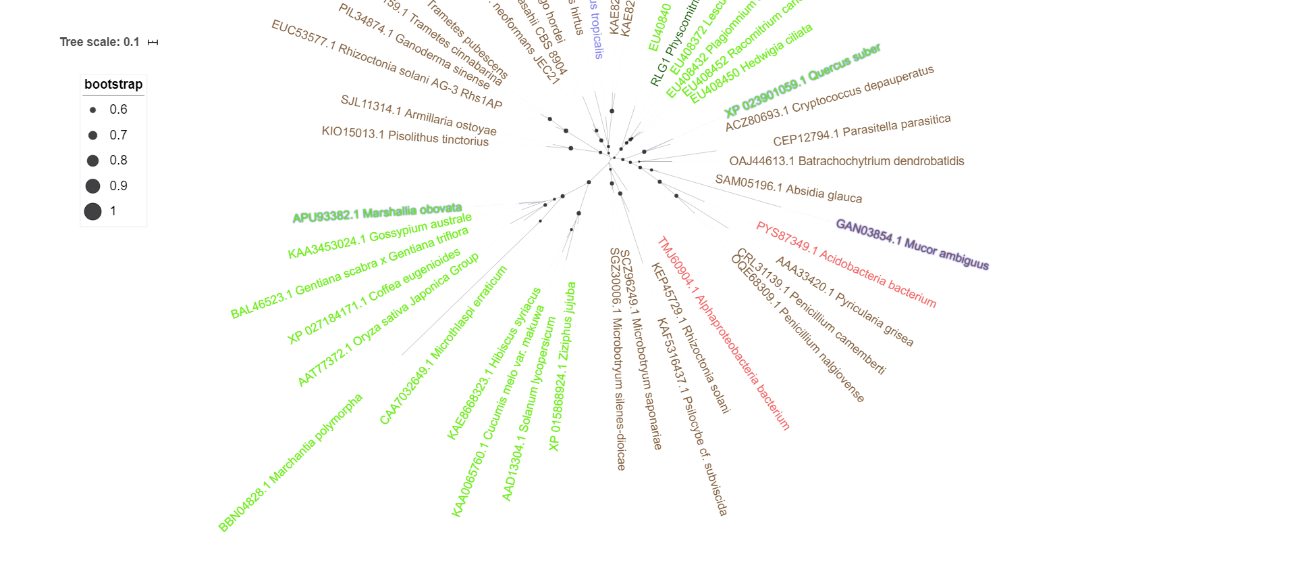

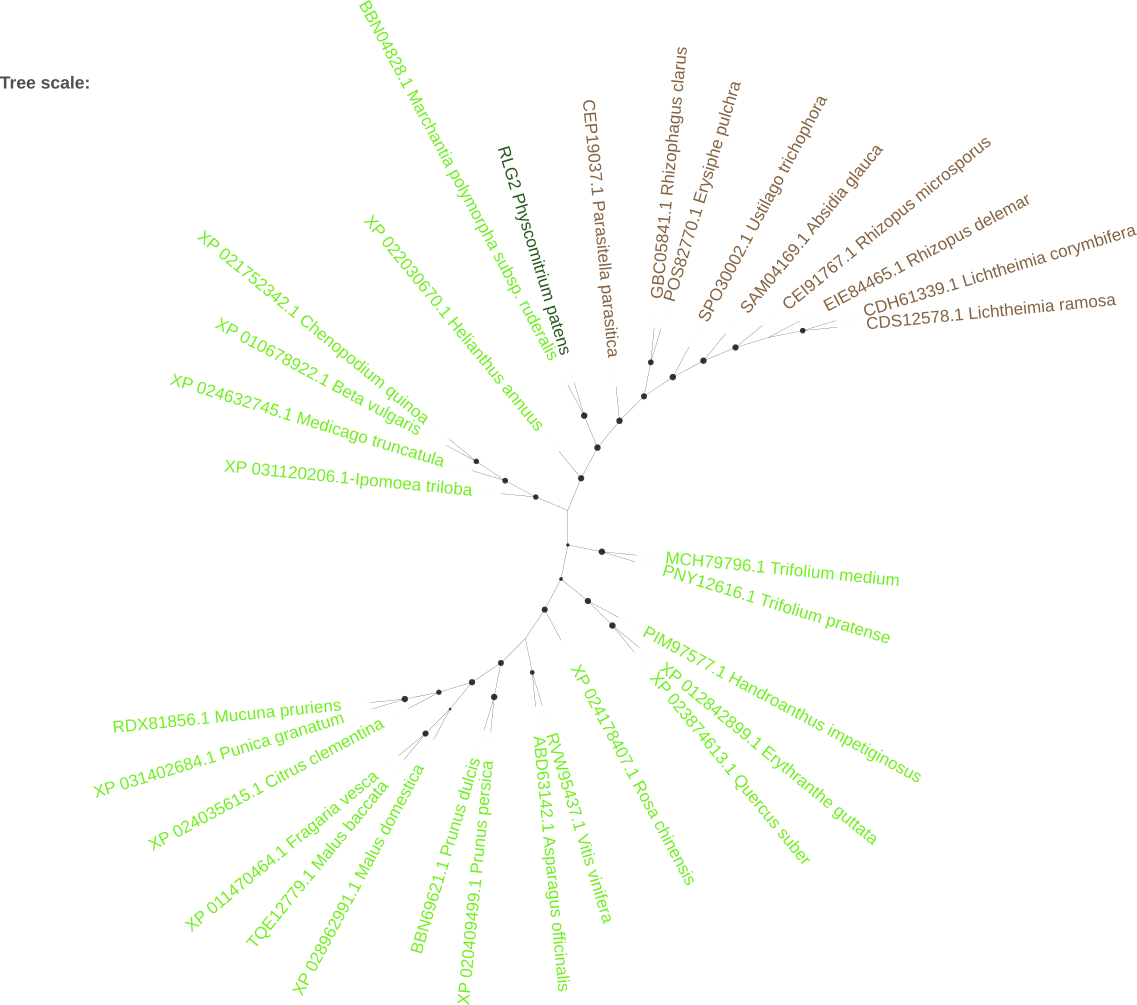


**Supplementary Figure 5: Phylogenetic analysis of the Transposable elements RLG2 proteins with those potentially encoded by plant, fungal, animals and bacterial transposable elements elements.** *P. patens* sequences are shown in dark green, plant sequences in light green, fungal sequences in brown


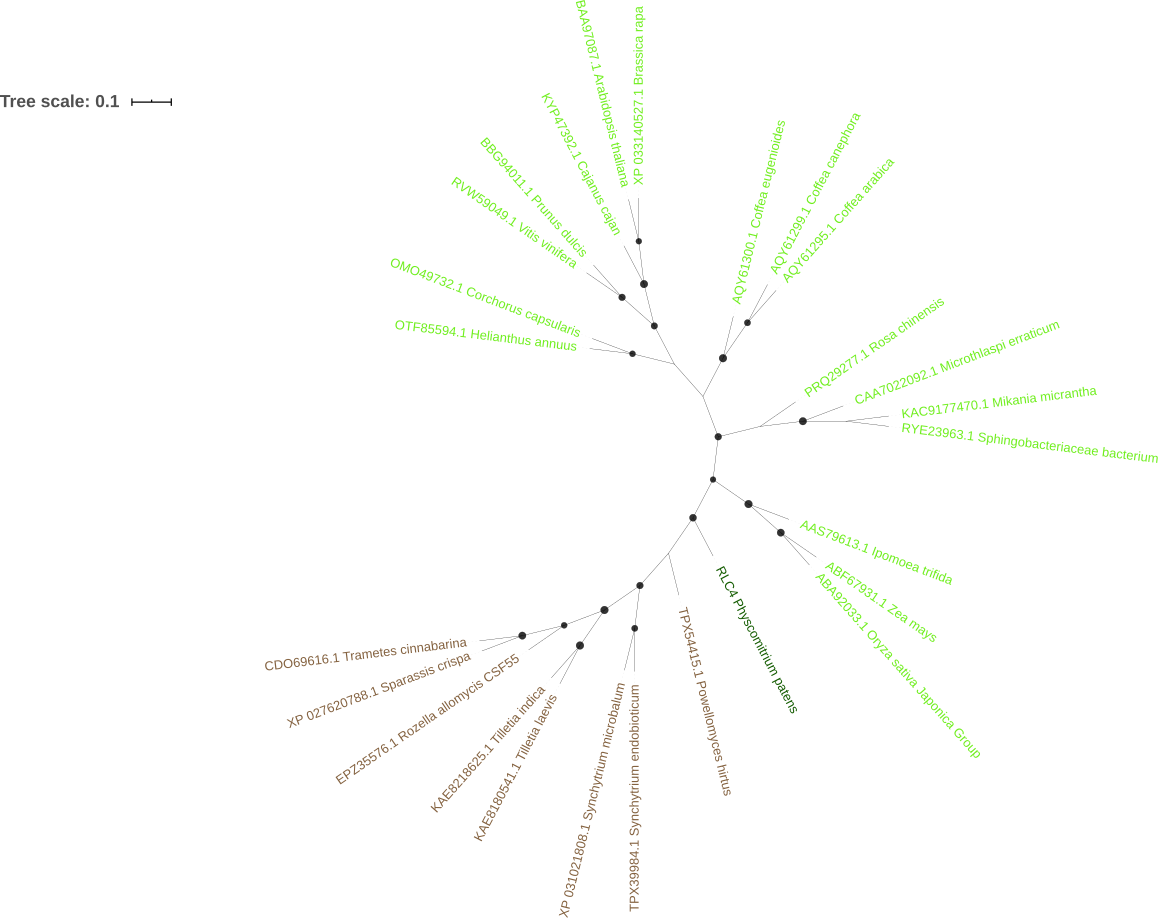

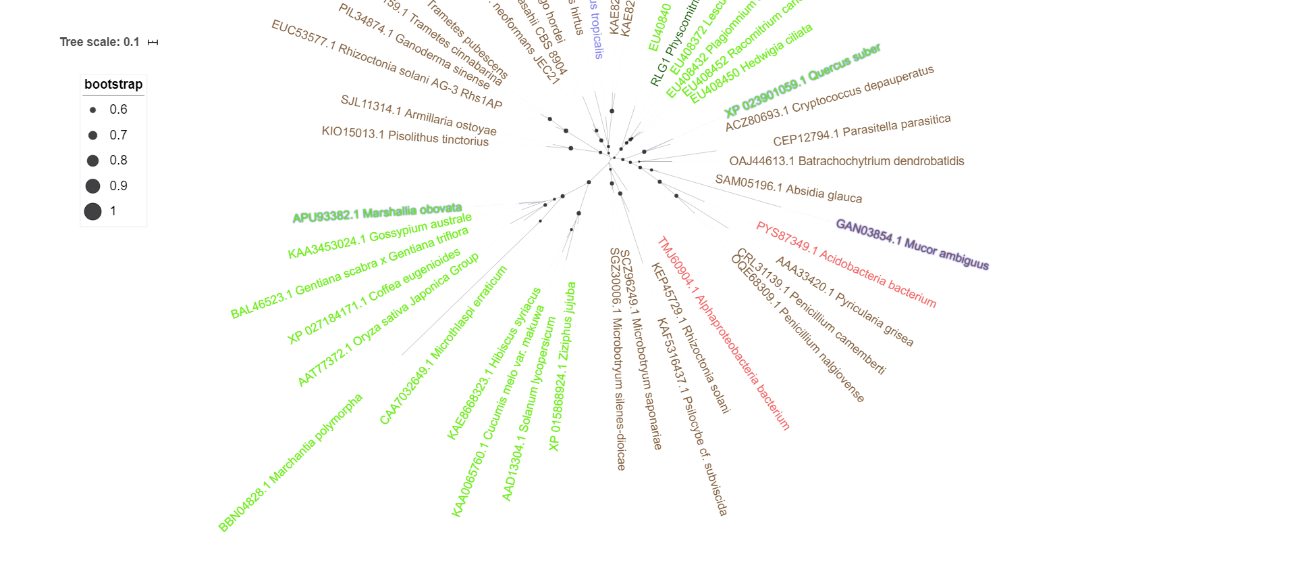


**Supplementary Figure 6: Phylogenetic analysis of the Transposable elements RLC4 proteins with those potentially encoded by plant, fungal, animals and bacterial transposable elements elements.** *P. patens* sequences are shown in dark green, plant sequences in light green, fungal sequences in brown


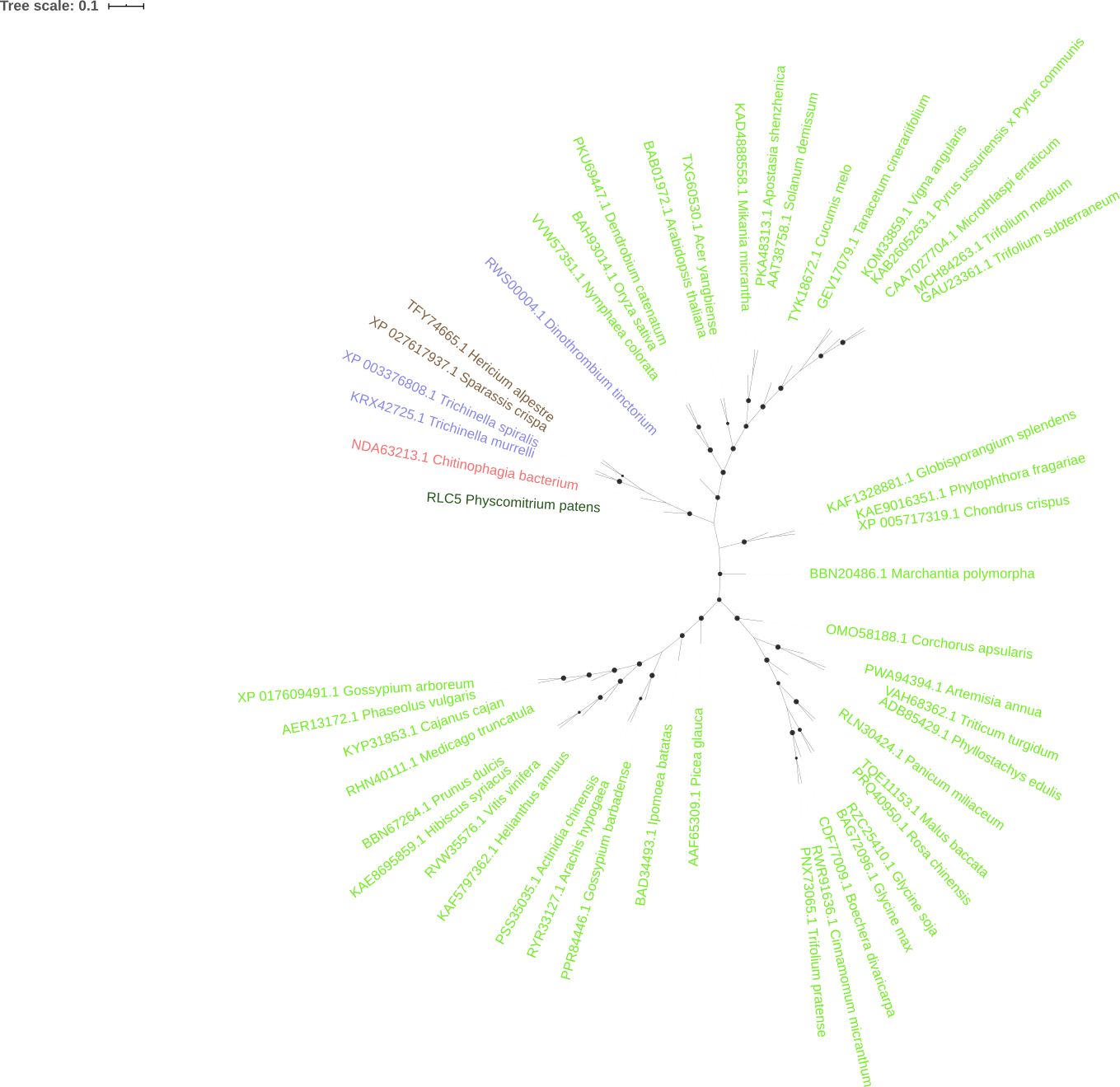

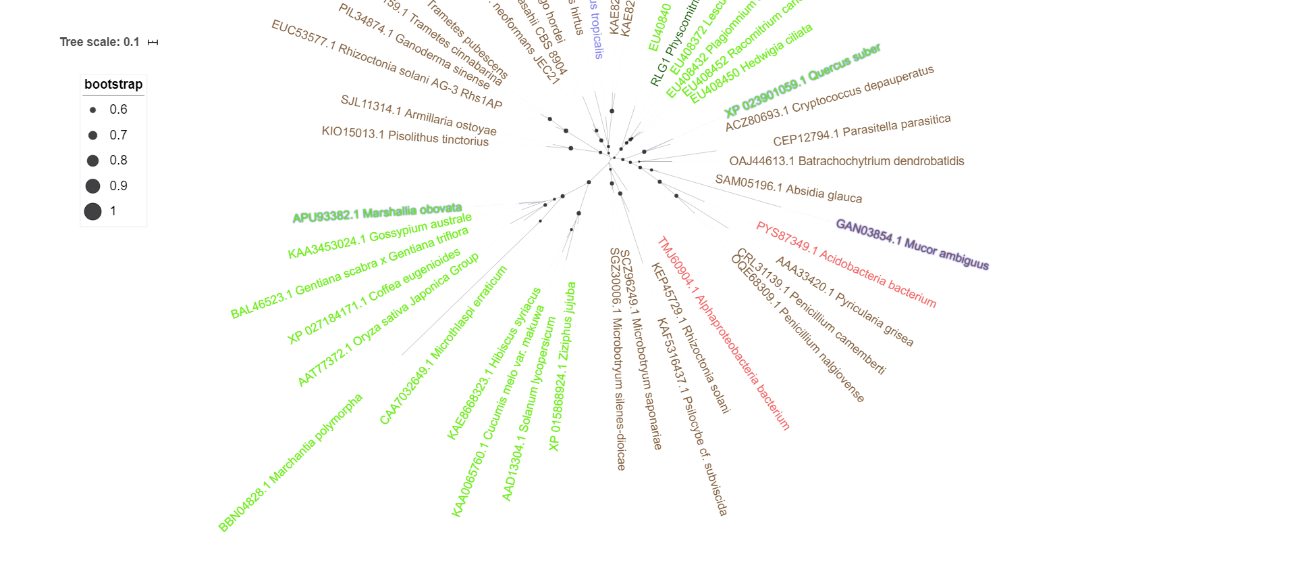


**Supplementary Figure 7: Phylogenetic analysis of the Transposable elements RLC5 proteins with those potentially encoded by plant, fungal, animals and bacterial transposable elements elements.** *P. patens* sequences are shown in dark green, plant sequences in light green, fungal sequences in brown, animal sequences in blue and bacterial sequences in red.


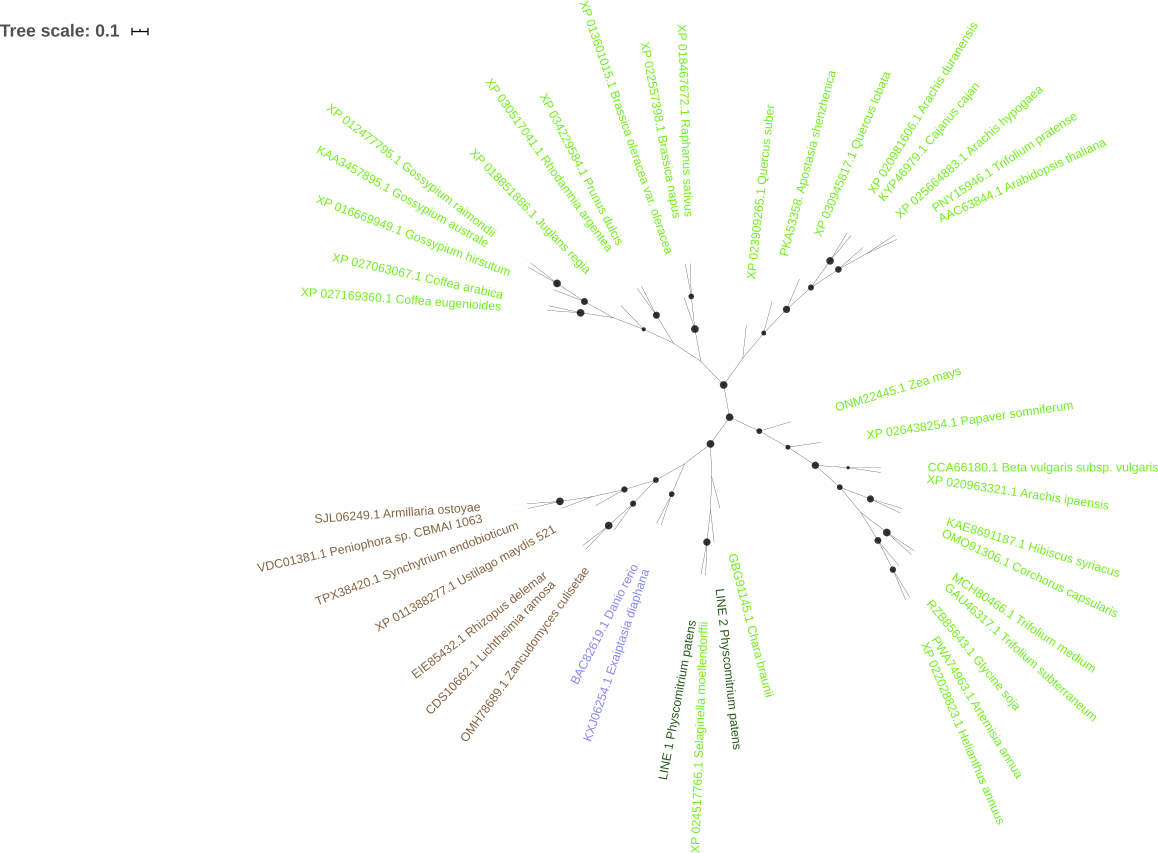

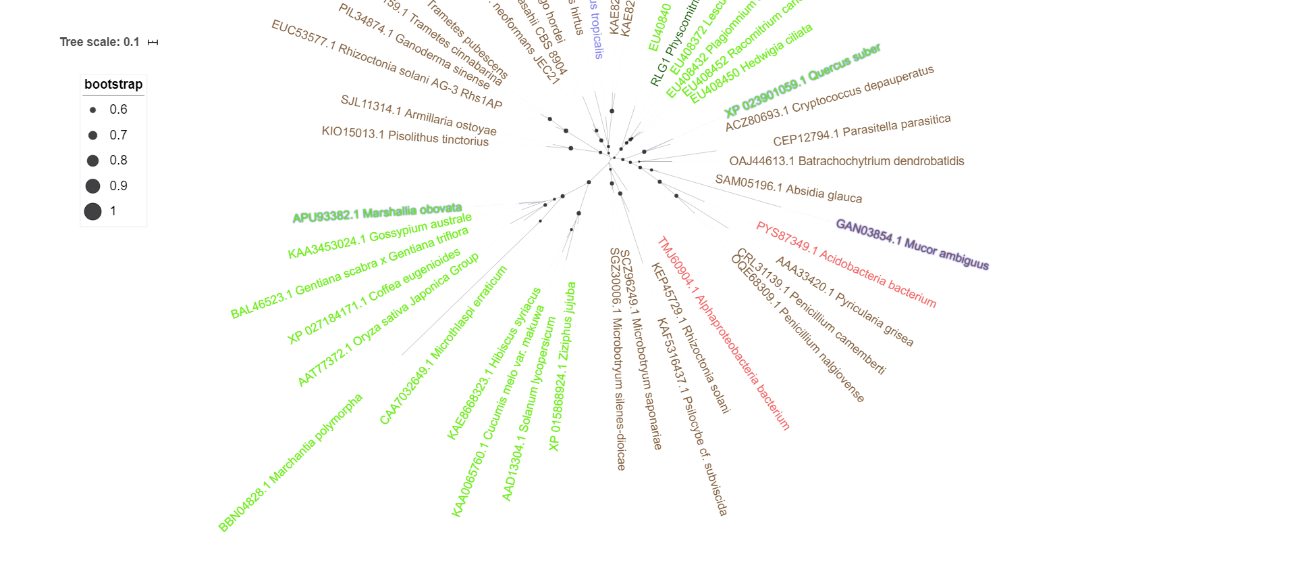


**Supplementary Figure 8: Phylogenetic analysis of the Transposable elements LINE-1 and LINE-2 proteins with those potentially encoded by plant, fungal, animals and bacterial transposable elements elements.** *P. patens* sequences are shown in dark green, plant sequences in light green, fungal sequences in brown and animal sequences in blue


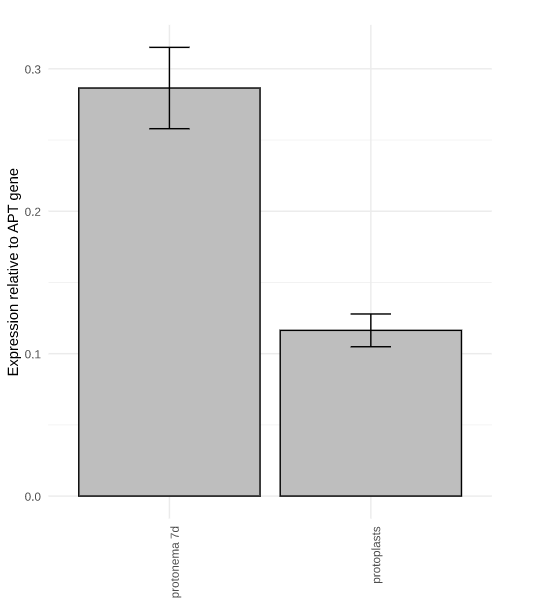


**Supplementary Figure 9:** qRT-PCR analysis of the expression of RLG1 in protonema grown for 7 days in BCDAT medium compared with the expression in protoplasts cells. The bars present the mean of three biological replicates. The standard deviation is shown.


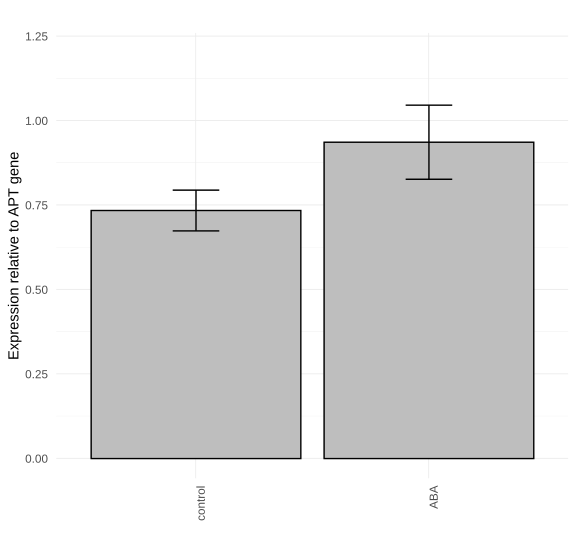


**Supplementary Figure 10: Induction of RLG2 expression by ABA.** qRT-PCR analysis of the RLG2 expression in protonema grown for 7 days in BCD medium (cotrol) and protonema grown for 6 days in BCD and one additional day in 50 µM ABA (ABA). The bars present the mean of three biological replicates. The standard deviation is shown.


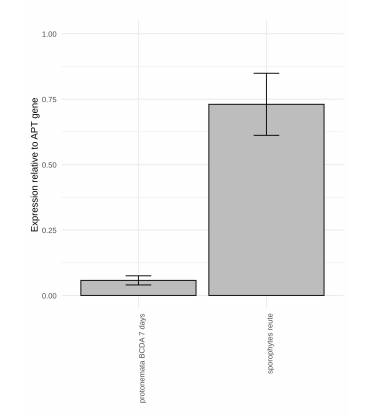


**Supplementary Figure 11:** Expression of tRLC5 elements in sporophytes. qRT-PCR analysis of the expression of tRLC5 elements in P. patens Reute sporophytes compared with its expression in protonema.


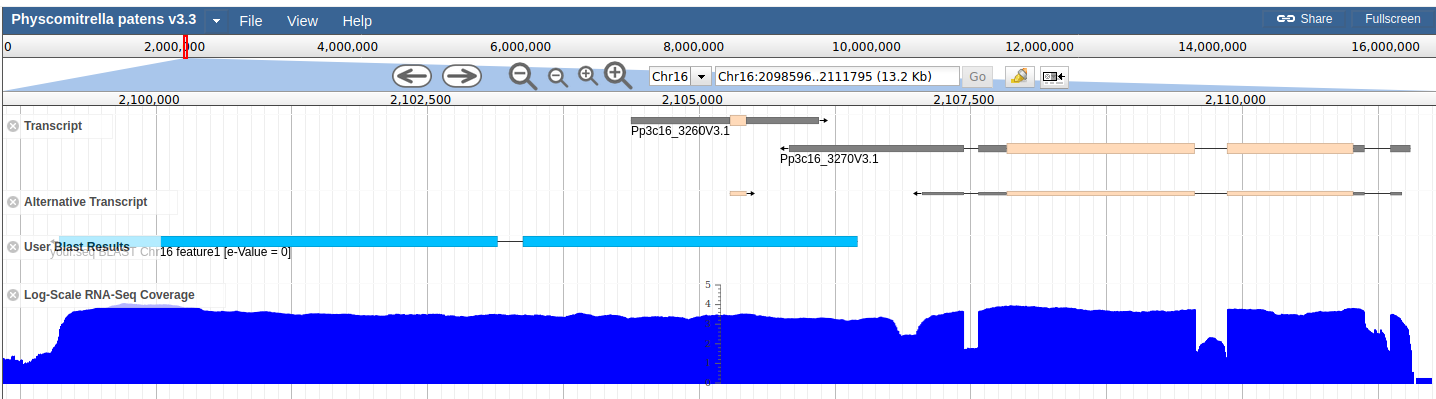


**Supplementary Figure 12:** **Expression of the LINE-2 inserted at the 3' of a gene and potentially transcribed as the result of read-through transcription.** Screenshot of the Phytozome genome browser showing from top to bottom, the Pp3c16_3270 predicted transcripts, the annotated alternative transcripts, the nBLAST best hit of the TRINITY_DN90_c0_g1_i9 assembly, and the log-scale RNA-Seq coverage of the genome region.


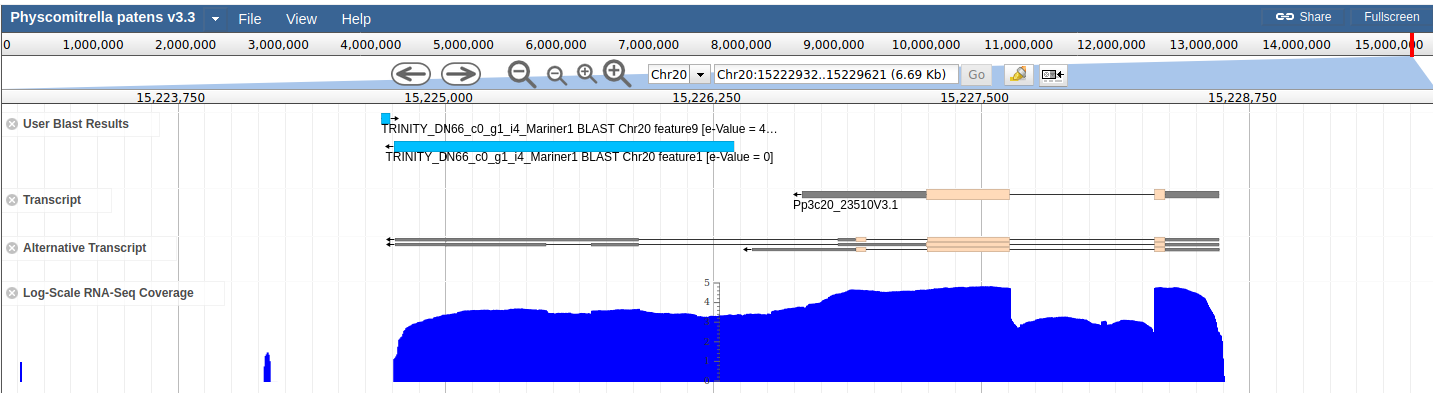


**Supplementary Figure 13:** **Expression of *PpTc1* as a result of read-through from the Pp3c20_23510 gene.** Screenshot of the Phytozome genome browser showing from top to bottom, the best hit of the TRINITY_DN66_c0_g1_i4_Pp_Tc1 assembly, the Pp3c20_23510 gene transcript, the alternative transcript of the gene track, and the log-scale RNA-Seq coverage of the genome region.


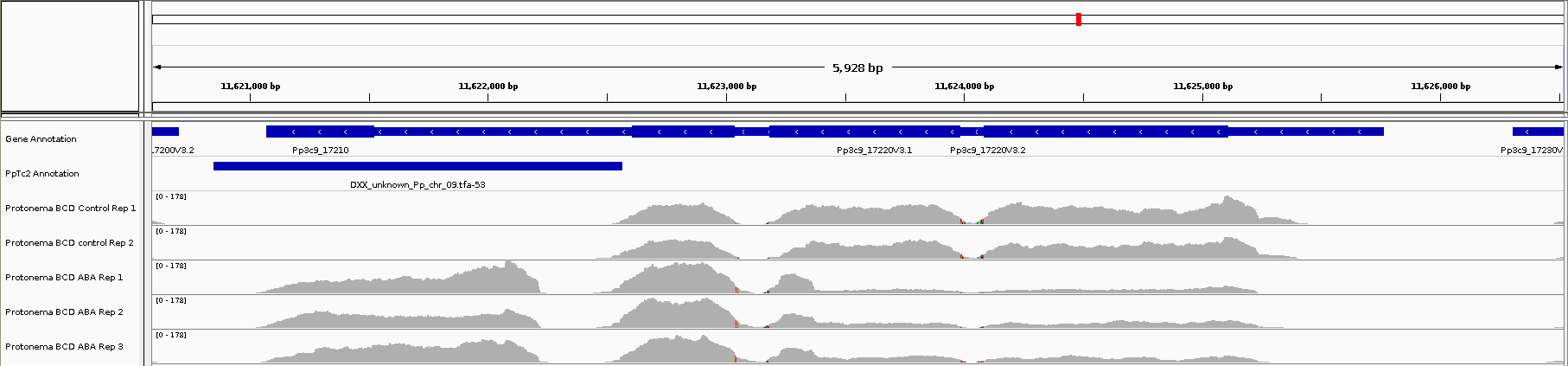


**Supplementary Figure 14:** **Expression of *PpTc2* and the Pp3c9_17210 gene located nearby.** Screenshot of the IGV genome browser showing from top to bottom, the annotation the Pp3c9_17210 gene, *PpTc2* annotation, RNA-Seq coverage of the genome region for two control protonema and 3 ABA-induced protonema samples.


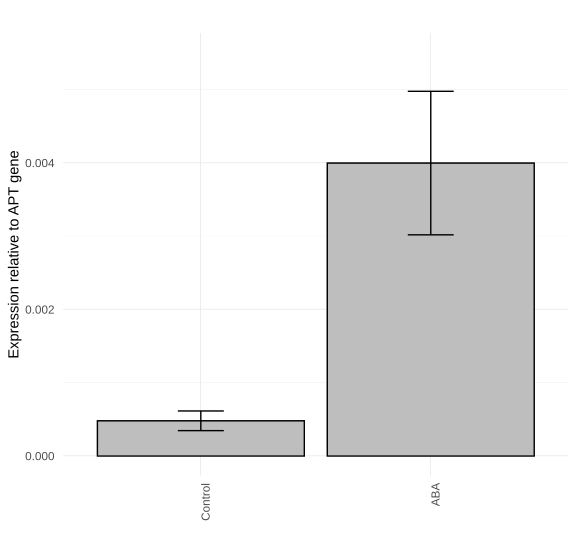


**Supplementary Figure 15: Induction of *PpTc2* expression by ABA.** qRT-PCR analysis of the *PpTc2* expression in protonema grown for 7 days in BCD medium (control) and protonema grown for 6 days in BCD and one additional day in 50 µM ABA (ABA).


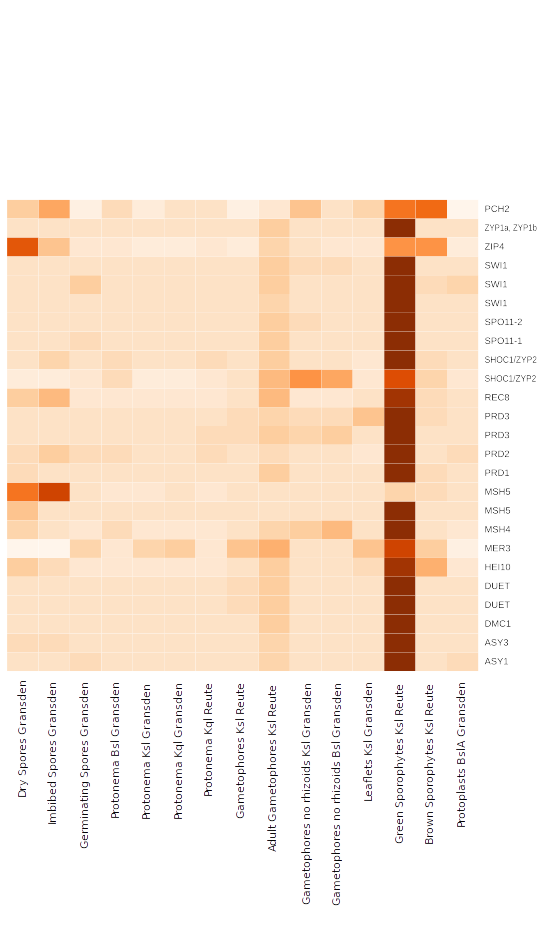


**Supplementary Figure 16: Expression of meiosis-specific genes in young sporophites.** Heat map of the expression of the meiosis-specific genes in different development conditions. The higher the expression, the darker the color.
